# An intervention strategy for improving residential environment and positive mental health among public housing tenants: rationale, design and methods of *Flash on my neighborhood!*

**DOI:** 10.1186/s12889-017-4730-9

**Published:** 2017-09-25

**Authors:** Janie Houle, Simon Coulombe, Stephanie Radziszewski, Xavier Leloup, Thomas Saïas, Juan Torres, Paul Morin

**Affiliations:** 1 Department of Psychology, Université du Québec à Montréal, C.P. 8888, succ. Centre-ville, Montréal, Québec H3C 3P8 Canada; 2 Department of Psychology, Wilfrid Laurier University, Waterloo, Canada; 3 Institut national de la recherche scientifique, Centre Urbanisation Culture Société, Montreal, Canada; 4 Urban planning, Université de Montréal, Montréal, Canada; 5 Social Work, Université de Sherbrooke, Sherbrooke, Canada

**Keywords:** Place-based intervention strategy, Positive mental health, Empowerment intervention, Residential environment, Public housing

## Abstract

**Background:**

In Canada, public housing programs are an important part of governmental strategies to fight poverty and public exclusion. The *Flash on my neighborhood!* project is a four-year multiphase community-based participatory action research strategy currently implemented in six public housing developments (*n* = 1009 households) across the province of Québec, Canada. The goal is to reduce the mental health disparities faced by these public housing tenants compared to the general population, while identifying which environmental and policy changes are needed to turn public housing settings into healthier environments.

**Methods:**

The protocol involves three successive, interconnected phases: 1) Strengths and needs assessment, including community outreach and recruitment of tenants to collaborate as peer researchers, an exploratory qualitative component (photovoice), a systematic neighborhood observation, and a household survey; 2) Action plan development, including a community forum and interactive capacity-building and discussion sessions; 3) Action plan implementation and monitoring. The entire intervention is evaluated using a mixed-method design, framed within a multiple case study perspective. Throughout the project and particularly in the evaluation phase, data will be collected to record a) contextual factors (tenants’ previous experience of participation, history of public housing development, etc.); b) activities that took place and elements from the action plan that were implemented; and c) short- and medium-term outcomes (objective and perceived improvements in the quality of the residential setting, both physically and in terms of mental health and social capital).

**Discussion:**

The study will provide unprecedented evidence-based information on the key ingredients of a collective intervention process associated with the increased collective empowerment and positive mental health of public housing tenants.

## Background

The ultimate goal of the intervention discussed in this manuscript, *Flash on my neighborhood!*, is to reduce mental health inequalities faced by public housing tenants. In Canada, the public housing program is an important component of governmental strategies to fight against poverty and social exclusion. In the province of Quebec, households who live in public housing developments pay only 25% of their income as rent, the remaining being funded by the government. There were close to 74,000 households benefiting from this program in Québec in 2011 [1]. Differently than in other countries, public housing is considered to be a residual housing option in Canada. Only people living in poverty have access to this program. Furthermore, in Québec, the largest proportion of public housing tenants is made of elderly people, mostly living in buildings that are reserved to this population.

Even if they have access to an affordable dwelling, public housing tenants suffer from a higher burden of physical and mental disease and have lower well-being and life expectancy than the general population [2–6]. Public housing tenants are a vulnerable population in the sense intended by Frohlich and Potvin (p. 218): “a subgroup or subpopulation who, because of shared social characteristics, is at higher risk of risks. The notion of vulnerable populations refers to groups who, because of their position in the social strata, are commonly exposed to contextual conditions that distinguish them from the rest of the population.” [7] Accessibility to public housing is restricted to low-income people (mostly living on social assistance benefits) presenting cumulative vulnerabilities, such as single parenthood, physical and mental disorders, recent immigration, large family size, and exclusion from the job market, or precarious and low paid jobs. Limiting public housing to a highly vulnerable portion of the population increases tenants’ stigmatization by other citizens [8–10].

A focus group study conducted among public housing tenants (*n* = 28) in Baltimore showed how social isolation was pervasive in this residential environment, possibly influenced by the lack of trust between neighbors, and how tenants viewed increasing neighborhood social capital as a promising strategy to their improve well-being [11]. Research suggests that the built environment is also often problematic, as public housing generally consists of multi-storey buildings^1^ that are poorly soundproofed and ventilated, over-crowded, and regularly infested by vermin [8, 11–13].

Social and physical aspects of the residential environment, including the apartment, building, and neighborhood, are important determinants of physical and mental health [14, 15], especially for people living in poverty who are generally limited in their daily mobility due to financial constraints and exclusion from the job market [16, 17]. They spend an important proportion of their time in their house and neighborhood [18], and are thus considered a locally-dependent group [19]. Public housing settings have the potential to be health-promoting environments that contribute to lessening social health inequalities instead of increasing them. However, this potential is often not fully exploited as public housing programs are often limited to providing shelter, without fully considering all the aspects that can contribute to (or hinder) tenants’ positive mental health. The present study focuses on the mental health-promoting potential of public housing settings, by implementing and evaluating an empowerment-based participatory intervention that aims to enhance public housing tenants’ mental health by engaging their community in a process focused on improving their residential environment.

Social health inequalities have been described extensively and comprehensively [20, 21]. A social gradient is observed for almost all diseases and causes of death, including mental disorders [22–25]. Empirical evidence suggests that socioeconomic status influences health through the activation of the chronic social stress response, which creates a detrimental allostatic load, linked to a variety of diseases, including cardiovascular disease, diabetes, cancer, and depression [26–32]. Several decades of research have repeatedly identified control over one’s life and social capital as important mediators of the physiological impact of chronic social stress [33, 34]. Social capital is defined by Bourdieu (p. 248–249) as “the aggregate of the actual or potential resources which are linked to possession of a durable network of more or less institutionalized relationships of mutual acquaintance or recognition.” [35] Based on this body of research, it has been hypothesized that, in order to reduce social health inequalities, interventions should increase the level of control people from low social status have over their life, as well as their social capital [15, 32, 36, 37].

Previous public health intervention research in public housing settings primarily focused on the reduction of risk factors for physical diseases, such as smoking, physical inactivity, and insufficient fruit and vegetable intake [38–42]. Until now, mental health has been neglected by interventions designed to reduce social health inequalities. Among the 319 studies identified in a recent systematic review of community engagement interventions to reduce health inequalities, only three focused on mental health, and none of those on interventions being implemented in a public housing setting [43]. Improving mental health among public housing tenants should be prioritized, since a person who enjoys good mental health “realizes his or her own potential, can cope with the normal stresses of life, […] and is able to make a contribution to her or his community”. [44] Furthermore, people in good mental health are more prone to adopt a healthy lifestyle [45–48] and are likely to be more successful in quitting bad habits such as smoking [49].

Mental health is more than the absence of illness, it also includes a state of well-being. Indeed, empirical evidence suggests that mental illness and positive mental health are two distinct constructs (although they are somewhat correlated) [50, 51], and that improvement in positive mental health predicts a decline in mental illness over time [52]. Positive mental health includes two aspects of well-being: 1) a hedonic aspect, which relates to having pleasurable experiences, such as being satisfied with one’s life and feeling more positive than negative emotions; and 2) an eudaimonic aspect, which refers to feeling that one’s life is meaningful, and to thriving through fulfilling relationships and engagement that contributes to society [50, 53]. High positive mental health, particularly positive relations with others and purposeful engagement, has been found to be related to lower biological markers of chronic stress, all-cause mortality, cardiovascular events, and stroke [54–57]. On the other hand, material deprivation, social exclusion, and poor quality of the residential environment have been negatively associated with aspects of positive mental health [58–60].

The reality of social health inequalities has been well established, and scientific inquiry has uncovered several mechanisms through which social determinants contribute to people’s (ill-)health (e.g., via control over one’s life and social capital); more research is now needed on how to influence these mechanisms to reduce inequalities [61, 62], particularly among vulnerable populations such as public housing tenants. Frohlich and Potvin [7] have suggested that a vulnerable population approach to intervention should have two characteristics: 1) be intersectoral, because social determinants of health are often located outside the health sector; and 2) involve the participation of the targeted population. Thus, any efforts toward reducing social health inequalities for public housing tenants should involve tenants themselves in describing the issue and in conceptualizing, implementing, and evaluating the intervention.

*Flash on my neighborhood!*, the intervention whose implementation and evaluation protocol is described in this article, incorporates these state-of-the art principles in a four-year multiphase community-based participatory action research strategy. Participatory action research is increasingly popular, but few empirical studies have focused on its real capacity to produce social change or the mechanisms through which it can generate its effects [63, 64]. *Flash on my neighborhood!* aims to improve the positive mental health of public housing tenants (thereby reducing the social inequalities they face compared to the general population), through focusing on social determinants, such as environmental and policy changes that are needed to make public housing settings healthier environments. *Flash on my neighborhood!* is a place-based intervention that gets public housing tenants involved in critically assessing their own residential environment (its strengths and weaknesses, how it promotes or hinders their well-being), developing an action plan to improve the situation, and then in implementing and evaluating that plan.

*Flash on my neighborhood!* uses a collective empowerment strategy to foster person–environment congruence [65]. Collective empowerment is defined as “a united and systematic effort by a group to gain control over and improve their aggregated lives by defining problems, assets, solutions, and the processes by which change can occur, and by building individual and collective capacity that can energize the power and knowledge existing within the assembly” (p. 213) [66]. In line with this definition, a collective empowerment strategy should improve tenants’ control over decisions that affect their life as well as their social capital.

Other place-based interventions targeting deprived neighborhoods have been examined empirically, but were found to be “unfocused, unsubstantial and short-term” [67]. In *Flash on my neighborhood!*, the four-year collective effort of public housing tenants will be focused on a specific target: improving person–environment congruence [65], which represents the adequacy between their needs, capabilities, and aspirations on the one hand, and the residential environment’s resources, demands, and opportunities on the other. Based on Horelli’s model for locally-dependent populations [19], we conceptualized the residential environmental as having four distinct types of structures that can support congruence: 1) physical structures, or the built and natural environment (e.g., buildings, roads, and green spaces); 2) functional structures, which represent the services available, such as transportation, stores, leisure centers; 3) participatory structures, which refer to opportunities to participate in the political and social life of the community, for instance through volunteering, or to develop abilities through involvement in specific projects; and 4) sociocultural structures, which relate to the sense of community, mutual aid between neighbors, and the shared culture of the group.

**Fig. 1:**
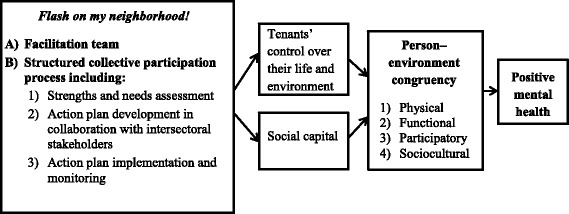
Summary of the *Flash on my neighborhood!* logic model

Figure 1 presents the logic model of *Flash on my neighborhood!*. As shown in the model, the strategy involves a facilitation team accompanying public housing residents through a structured collective participation process, including a strengths and needs assessment, and the development, implementation, and monitoring of an action plan to address the needs for improvement. It is theorized that these activities will promote tenants’ control over their environment as well as their social capital, making them more likely to act towards improving the congruence of their environment, which in turn should promote their positive mental health.

### Study aims

Implementation studies can provide “extremely relevant information that can be used in other settings, for the improvement in the delivery of other types of interventions” (p. 115) [62]. In that perspective, the aim of the present protocol is to: 1) evaluate the implementation of *Flash on my neighborhood!* in six public housing developments; and 2) determine the perceived effects on person–environment congruency and positive mental health among public housing tenants.

### Research questions

The study aims to answer six questions: 1) How do contextual factors influence the intervention’s implementation? 2) Which improvements in the residential environment are prioritized by public housing tenants? 3) Which planned improvements in the residential environment succeeded and which failed? 4) What are the contextual factors (e.g., urban vs. rural area, level of cultural diversity in the public housing setting, etc.) that played a role in the success or failure of the planned improvements? 5) Did the intervention improve tenants’ perceived control over their environment and social capital? 6) Did tenants perceive improved person–environment congruency and positive mental health as a result of the intervention?

## Methods

### Design

The study uses a prospective, multiple case study design [68, 69], which allows for an in-depth investigation of complex changes in real contexts. Each unit of analysis (case) is composed of a public housing development, its tenants, the staff and managers of the city’s housing agency, as well as stakeholders (staff, managers, and decision-makers) from community organizations and the municipal government of the areas in which the project is implemented. A multiple case study design is essential to evaluate a complex intervention such as *Flash on my neighborhood!* because it allows researchers to document the intervention’s general functioning while also investigating how the contextual characteristics of the setting in each case influence its implementation [68]. Using the same method in multiple cases leads to an enhanced understanding of the phenomenon, and also increases the validity and generalizability of the results [70–72]. Similarities and differences are recorded, to develop significant patterns from the data [73].

### Settings

The study takes place in six public housing developments in the province of Quebec, Canada: Montreal, Saint-Hyacinthe, Cowansville, Lévis, Gatineau, and Trois-Rivières. These six cases were selected through intentional sampling to maximize their diversity in terms of 1) city size (number of citizens), 2) size of the city’s housing agency, 3) number of dwellings in the development, 4) type of tenants (families, one-person households, seniors or mixed), 5) building characteristics (high-rises >4 storeys, buildings ≤4 storeys, townhouses), and 6) cultural diversity (see Table 1).

**Table 1:** Characteristics of the settings

City	Population	Size of the city’s housing agency (units)	Number of dwellings	Tenants	Type of buildings
Montreal	> 1,500,000	30,385	177	Families	1 high-rise, 3 buildings, 5 townhouses
St-Hyacinthe	± 53,000	668	119	Families	3 buildings, 48 townhouses
Cowansville	±13,000	198	98	Mixed	9 buildings, 10 townhouses
Lévis	± 140,000	1263	84	Single-person household	3 buildings
Gatineau	± 260,000	3597	398	Mixed	3 high-rises
Trois-Rivières	± 130,000	1864	133	Seniors	1 high-rise

Buildings are ≤4 storeys, while high-rises are >4 storeys

#### *Flash on my neighborhood!* Intervention

For tenants to develop control over the changes that *they* perceived as important, we chose to standardize the process rather than the content of the intervention [74, 75]. The intervention involves a facilitation team and a structured collective process including three phases: 1) Strengths and needs assessment; 2) Action plan development; and 3) Action plan implementation and monitoring.

#### Facilitation team

Each site is assisted by a facilitation team composed of two graduate students (in community psychology, social work, or psychoeducation) and one undergraduate student (total *n* = 18), supervised by the principal investigator (PI). The facilitation teams have to follow a structured protocol for each of the three steps to assure a rigorous implementation of the intervention and to enhance the reliability of the results in a multiple case study [69, 72, 76]. The facilitation team’s role is to support tenants’ capacity-building at each step of the process. They model how to organize and moderate a meeting and how to solve interpersonal conflicts. They also provide tenants practical tools for long-term use beyond the intervention, such as factsheets that show how to set realistic and achievable objectives and how to make an action plan. The facilitation team uses a gradual process of empowerment in which they provide more leadership in the first stages of the project, but transfer more and more responsibilities to tenants as it progresses. At the end of the project, tenants should be more autonomous in organizing citizen participation initiatives in their setting.

#### Strengths and needs assessment

This step aims to describe the assets and improvement needs in the residential environment of each setting. Four sequential activities will be performed for that purpose: a) recruitment and training of tenant researchers; b) a photovoice project (see [77] for a description of this activity in the Montreal setting); c) systematic neighborhood observation with a grid (see [78] for a description of this activity in the Montreal setting); and d) a household survey.

- In each site, eight to twelve public housing tenants are recruited and trained to act as peer researchers, or what we call *tenant researchers*. The involvement of peer researchers is increasingly popular in public health [79], because it promotes capacity-building and empowerment of community members and increases data validity [79–81]. In the present study, various means are used to recruit tenant researchers: written invitations to each household, posters, outreach by the tenants’ association, and information meetings. Following best practices [79], tenant researchers will participate significantly in data collection, data analysis, data interpretation, and knowledge mobilization, as well as to in action plan development and implementation. They receive appropriate training (research ethics, theories of mental health and environment, photography). Over time, they develop other capabilities, such as content analysis, and writing and communication skills. A stipend of $20 is given to each tenant researchers for their participation in the *strengths and needs assessment* activities.
- In the photovoice activity, the tenant researchers are accompanied by the facilitation team through eight weekly meetings. The first two meetings consist in training on research ethics and photography, and an explanation of the study’s concepts. Over the next four weeks, prior to meetings three to six, the tenant researchers take pictures of their residential environment, selecting two of them to bring to the following meeting for group discussion. These audio-recorded discussions help the group identify themes under which to classify the pictures. In the two final meetings, the tenant researchers finalize the thematic analysis, write captions for each of their pictures, and collaborate on organizing a public exhibit of their work. At the official opening for the exhibit, community stakeholders (such as the city mayor or housing agency director) are invited to acknowledge the work and perspectives of the tenant researchers. The photovoice process seeks to engage members of a community in collecting data about the community’s strengths and weaknesses, to promote critical knowledge and dialogue concerning the community’s important issues, and to reach decision-makers in order to produce social change [82].
- During the systematic neighborhood observation activity, the tenant researchers assign a score of 0 (*very unsatisfying*) to 4 (*very satisfying*) to 64 elements in their residential environment based on their observation and appreciation of the quality of those elements. A two-member facilitation team accompanies the group through five weekly meetings. In the first meeting, a neighborhood observation grid (see [78]) is presented, and the tenant researchers form teams of two or three. Before each of the following four meetings, these small teams walk through their residential environment to complete the grid, section by section: 1) Public housing development; 2) Streets and buildings in the neighborhood; 3) Green spaces in the neighborhood; 4) Interpersonal relationships in the neighborhood and 5) Shops, community organizations, and services. During the group meetings, the teams present their score for each item as well as justifications for their choice. Afterwards, the group discusses to agree on a single collective score for each item. A facilitation technique adapted from the Canadian Institutes of Health Research grant evaluation technique is used to prevent discussions from escalating. Each tenant researcher can confidentially allocate a parallel score equivalent to 0.5 points over or under the collective score. This ensures that every tenant researcher feels that their opinion is respected, while making the collective decision-making process easier.
- In each household, one adult is invited to complete a survey on their residential environment and positive mental health. The survey includes items on positive mental health (a validated scale, [83]), person–environment fit (a questionnaire designed for the purpose of the project; validation underway), individual control ([84]), collective empowerment (inspired by existing items [85, 86]), and sense of community, (Saïas T, Loomis C, Beck F: Validation of the French Version of the Brief Sense of Community Index, submitted) as well as open-ended questions on tenants’ talents, passions, and projects that contribute to their well-being. Tenant researchers and/or the tenants’ associations are invited to design their own questions (e.g. about tenants’ willingness to participate in different activities) in addition to the standardized scales, to inform their activities and the next phase of the project (action plan). The one-hour survey is verbally administered by a university research assistant at the participants’ home or in a quiet confidential space, as participants prefer. Each participant receives a $10 compensation for their time. Extensive recruitment efforts are made during the data collection period. For example, tenant researchers help distribute an invitation to every household. They also help publicize the survey through ads posted in the public housing development.

#### Action plan development

Three sequential activities, facilitated by two research assistants and occasionally the PI, are organized to develop a one-year action plan. First, a half-day *collective forum* is organized by the tenant researchers. This event aims to share the results of the *strengths and needs assessment* with the other tenants and community stakeholders, and to identify the issues that will be addressed in the upcoming year, implementing principles from the participative urbanism perspective [87, 88]. Tenant researchers have complete control over the organization of the forum: they choose the content, they write and distribute the invitations, they organize the activities for adults and children during the event, etc. They are encouraged to ask other tenants to volunteer and to use creative strategies to maximize the participation rate to the *collective forum* (such as participation prizes, entertainment for children, free snacks and beverages, music). Each hour of volunteering is formally recognized through citizen participation certificates that mention how many hours the person has invested and what kind of tasks were accomplished. These certificates not only serve to underline the value of tenants’ contributions, but also to provide a record of the skills that they have developed through the project.

During the forum, tenant researchers present the four or five main themes that have emerged from the strengths and needs assessment phase, as identified in collaboration with the research team after having analyzed the results. Themes are then discussed in small groups between attendees, facilitated by a university student, with the goal of engaging attendees in interpreting the results related to the theme and in suggesting actions that should be implemented to improve the residential environment. Each small group is invited to share their action ideas with the larger group, which then votes to identify priority actions for each theme.

After the forum, the second activity consists in an interactive session given by the principal investigator about basic principles for producing change (inspired by [89]). Finally, the third activity consists of two meetings facilitated by the research team to elaborate the action plan with tenants, based on the priorities identified during the forum and the principles learned during the interactive session. Several strategies are used to involve more tenants in the action plan development activity, including reaching out to every tenant who attended the forum and sending personalized invitation letters to every tenant who indicated in the survey that they were willing to share their talents and passion with their neighbors.

The action plan describes specific objectives related to each of the two top priorities for every theme, manageable steps to follow to achieve those objectives, deadlines for each step, as well as resources and partners that could be helpful and potential pitfalls to avoid. Before the action plan is launched, it is presented by the tenants, to the housing agency employees and managers, as well as to other relevant community stakeholders to collect their comments and suggestions. However, the action plan belongs to the tenants, with the housing agency and other stakeholders being viewed as potential allies in its realization.

#### Action plan implementation and monitoring

The next phase is the implementation of the action plan over a 12-month period. During that time, monthly meetings are organized with the tenants to follow up on the previous month’s achievements and to share the tasks planned for the coming month. Progress is celebrated, problems are discussed, and the plan is adjusted as needed. The community’s progress is reviewed regularly by collecting objective and subjective information from participating tenants and stakeholders on four indicators: 1) tenants’ involvement in their setting (number of people involved and number of hours devoted to the project); 2) quality of the social relationships among the tenants; 3) tangible improvements in the residential settings related to the project; 4) relationships with community partners and allies. As time goes by, tasks initially performed by the facilitation team (e.g., organizing meetings, etc.) are progressively entrusted to the tenants and their allies to encourage them to become autonomous by the end of the project.

### Intervention evaluation

The study has received approval from the Ethics board of the Université du Québec à Montréal. A mixed-method evaluation was chosen for data collection and analysis. One strength of multiple case studies is the use of multiple data sources, which allows for triangulation and identifying potential convergence among the results [69].

To that end, the evaluation includes data from questionnaires, qualitative interviews, the research team’s observations (e.g., regular journal entries by facilitators), and review of documents, in order to capture: a) contextual factors (tenants’ previous experience of participation; history of the public housing development, etc.), b) activities that take place and priorities from the action plan that are implemented, and c) achievement of short- and mid-term outcomes (objective and perceived improvements in the quality of the residential setting, both physically and in terms of mental health and social capital).

Each tenant researcher completes questionnaires at the beginning of the photovoice process (pre-test) as well as after the public exhibit (three to four months later; post-test) to evaluate their self-reported positive mental health [83], personal control [84], collective empowerment (inspired by existing items [85, 86]), and sense of community (Saïas T, Loomis C, Beck F: Validation of the French Version of the Brief Sense of Community Index, submitted). Socio-demographic characteristics are collected as well. The post-test also includes a scale on perceived group dynamics, including items inspired from an existing scale from Moos [90, 91]. After the photovoice project, semi-structured qualitative interviews are conducted with tenant researchers to document their experience and the impact they perceive the project has had on them and on their neighbors up to that point. At the end of the action plan implementation phase, additional interviews will be conducted with tenants, as well as with housing agency managers and staff, to document the perceived impacts of the intervention on the tenants and on the residential environment.

Throughout the project, facilitation team members and the study coordinator complete a research journal entry after each interaction with tenants. They note their observations of the interactions with tenants, and between tenants and stakeholders. The meeting recordings, the pictures taken during the photovoice project, the scores obtained on the 64 environmental elements of the observation grid, the quantitative data collected in the household survey, the action plans, and meeting proceedings are all used to inform the evaluation.

### Data analysis

A cross-case analysis method will be followed [68, 71], consisting in four steps: 1) organizing each site’s data in a detailed case report; 2) using matrices and tables to identify key themes in each case; 3) examining the similarities and differences in these key themes to establish meaningful patterns; and 4) forming general explanations concerning the identified patterns. This type of analysis is a funnel process that serves to make sense out of a large quantity of information originating from multiple different sites. The ultimate goal is to generate explanations that will enhance our understanding of the phenomenon being studied. Each step represents an extension of the previous step, while the process remains iterative.

In the first step, each case is treated individually [68, 71]. The sociodemographic questionnaires, tenant researchers’ captions, interviews, meeting proceedings and recordings, and journal entries are all transcribed and uploaded along with the photos into the NVivo software application. A thematic analysis is performed in the following steps: gaining familiarity with the data; coding the data; looking for themes; revising the themes; defining and naming the themes [92, 93]. Quantitative data (survey answers, observation grid ratings) are analyzed using descriptive statistics, applying inferential statistical tests such as ANOVA to compare between the different implementation sites, and repeated-measures t-tests to compare pre- and post-test questionnaires.

Treating the data case by case, all of the data is analyzed and observations are recorded. Next, the results that are relevant to the research questions are coded systematically. When the coding is finished, a list of codes and associated quotes is created based on the qualitative data. This list is used to regroup the codes in more general themes. The themes are revised with reference to the list of codes and raw data to ensure that all the relevant data is included under the proposed themes. Finally, the themes are named and defined in terms of their contribution to the research questions [92, 93]. The results are collected in a case report. The six case reports each contain a description of the public housing development site under study, as well as the themes and quotes relevant to the research questions.

In the second stage, the researchers ensure they gain an understanding of each case in-depth before proceeding to a cross-case analysis [68]. To do so, each case report is read thoroughly. During this reading, a note matrix is completed, to pick out the elements that are key to answering the research questions [68, 71]. This step reduces the quantity of information that will be used to perform the cross-case analysis, while preserving the most important elements [68].

In the next step, once the matrices are completed, patterns of similarities and differences between the cases are highlighted. A comparison grid is filled out to better visualize these patterns [68, 71]. Certain themes will be found in all cases, while others will represent atypical themes, specific to one case but nevertheless worth reporting (Stake, 2006). The grid makes it possible to rapidly indicate the scope of each theme across the different cases while also presenting the context specific to each case.

In the final step, general explanations are formed using the patterns identified in the previous step. Particular attention is given to understanding how the patterns can best answer the research questions [68]. The comparison grid is reviewed to produce a summary grid containing the general explanations. This is an opportunity to reorganize, recombine, and elaborate on the general explanations. At this stage, efforts should be made to synthesize the findings [68]. The elements leading to the general explanations should be thoroughly evaluated. While revising the general explanations, it is important to reflect on the potential relationships between them. Are certain explanations linked? Does this add to their relevance, or does it give one explanation more weight?

### Methodological rigor

The methodological rigor of the multiple case study approach is sometimes called into question [69, 76]. However, several strategies can be put in place to ensure the study is highly rigorous [69].

#### Construct validity

Construct validity corresponds to the degree with which a study examines what it intends to examine [94]. Case studies are sometimes perceived as somewhat subjective [76]. To ensure construct validity in a case study, it is recommended to precisely define the concepts [69]. In the present study, residential environment is defined using a recognized theoretical model [19]. Also, triangulation of the data can reduce the subjectivity of interpretations [68, 69, 76]. When conducting a multiple case study, triangulation is performed within each case by using multiple data sources and across cases by verifying the convergence or divergence of results in different contexts [68].

#### Internal validity

Internal validity refers to the logical interpretation of data and therefore to the legitimacy of the study’s conclusions [76]. To ensure a correct interpretation of the data, a member-checking procedure [68] will take place at two crucial moments of the data analysis. The detailed case reports will be submitted to members of the facilitation team and tenant researchers for each case [70], who will validate the reports by suggesting modifications or clarifications. At the end of the analysis, when general explanations are formed, a synthesis will again be submitted to stakeholders from each case.

#### External validity

External validity corresponds to the degree to which a study’s results can be generalized [94]. Case studies do not aim to provide statistical generalization, which means that data from a case study does not lend itself to conclusions about an entire population [76]. The objective is rather analytical generalization, which is to generalize empirical observations to a build theory [69, 76]. In the present study, the empirical observations will contribute to a theory of the transaction between public housing tenants and their residential environment and how participatory interventions can help tenants take control to improve this transaction. A cross-case analysis including four to ten cases is considered a solid basis for analytical generalization [76]. Choosing cases with varied characteristics, as has been done in the present study, also promotes external validity [95].

#### Fidelity

Fidelity represents the possibility of replicating a study and obtaining the same results. In the present study, fidelity was strengthened by creating a multiple case study protocol to ensure a certain homogeneity in the intervention implementation and data collection methods across the six sites [69, 72, 76]. Furthermore, the entirety of the data, including an audit trail of all analyses performed, will be retained in a database, making it available for a later replication study [69, 72, 76].

## Discussion

*Flash on my neighborhood!* is the first population health intervention study that focuses on improving the positive mental health of public housing tenants. There is little knowledge concerning successful strategies for increasing vulnerable populations’ control over their environment and social capital, especially in public housing settings, where people are at high risk of experiencing social and psychological challenges. *Flash on my neighborhood!* provides an innovative opportunity to study a structured intervention process for developing public housing tenants’ collective empowerment in multiple settings, also contributing to a theoretical framework of contextual factors that facilitate or impede such an intervention process. As public housing authorities are generally relatively unfamiliar with tenant empowerment practices, they tend to form a highly hierarchical structure within which tenants have little real control over the choice of their dwelling and many aspects of their residential environment [96]. Thus, tenants are often captive of a stigmatized residential environment, bound by numerous rules (which they have not contributed to formulating), and denied direct contact with landlords and decision-makers.

By explicitly focusing on increasing residents’ control over their environment and social capital, *Flash on my neighborhood!* targets two crucial social determinants of health in order to contribute to an emerging “science of solutions” to reduce social health inequities [62]. The study will also make a significant methodological contribution through the use and validation of a person–environment congruency questionnaire and a systematic observation grid that are adapted to the public housing setting.

Finally, the results will also inform practitioners regarding public housing tenants’ priorities for improving their residential settings, and will provide participatory intervention strategies to turn such settings into environments that promote mental health. We will know more about the conditions to put in place to help these residents achieve their goals and aspirations, and thus their well-being. There are many public housing settings around the world; the results of this study will help policy-makers, managers, and staff design environments that promote tenants’ mental health, thereby reducing the inequities they face.

## Footnotes

1 Although the quality of the built environment is still perceived to be an issue (e.g., [97]), in Québec, Canada, multi-storey public housing buildings represent the exception rather than the rule.
